# Impact of gut microbiota and associated mechanisms on postprandial glucose levels in patients with diabetes

**DOI:** 10.2478/jtim-2023-0116

**Published:** 2023-12-20

**Authors:** Xinyuan Feng, Mingqun Deng, Lina Zhang, Qi Pan

**Affiliations:** Department of Endocrinology, Beijing Hospital, National Center of Gerontology, Institute of Geriatric Medicine, Beijing 100730 ,China; Graduate School of Peking Union Medical College, Chinese Academy of Medical Sciences, Beijing 100730, China

**Keywords:** gut microbiota, postprandial glucose, precision medicine, short-chain fatty acids, bile acids, trimethylamine N-oxide

## Abstract

Diabetes and its complications are serious medical and global burdens, often manifesting as postprandial hyperglycemia. In recent years, considerable research attention has focused on relationships between the gut microbiota and circulating postprandial glucose (PPG). Different population studies have suggested that PPG is closely related to the gut microbiota which may impact PPG via short-chain fatty acids (SCFAs), bile acids (BAs) and trimethylamine N-oxide (TMAO). Studies now show that gut microbiota models can predict PPG, with individualized nutrition intervention strategies used to regulate gut microbiota and improve glucose metabolism to facilitate the precision treatment of diabetes. However, few studies have been conducted in patients with diabetes. Therefore, little is known about the relationships between the gut microbiota and PPG in this cohort. Thus, more research is required to identify key gut microbiota and associated metabolites and pathways impacting PPG to provide potential therapeutic targets for PPG.

## Introduction

Diabetes is one of the most common chronic non-communicable diseases which threatens global human health.^[1, 2]^ The International Diabetes Federation (10^th^ Diabetes Atlas, 2021) reports that global disease prevalence, between the ages of 20 to 79 years, is estimated to be 10.5% (536.6 million adults),^[3]^ with disease-related health expenditure is estimated at 966 billion dollars and is expected to reach 10.54 trillion dollars by 2045.^[4]^ Diabetic nephropathy, diabetic retinopathy, cardiovascular events and other diabetes-related complications seriously affect the quality of life of patients, increasing the hospitalization rate and mortality of patients.^[5, 6, 7, 8, 9, 10, 11]^ In China, approximately 33% of diabetic outpatients achieve blood glucose targets.^[12]^ Blood glucose fluctuation, especially postprandial hyperglycemia, is closely related to diabetes complications.^[13]^ Glycemic control was assessed using the hemoglobin A1c (HbA1c) level.^[14]^ The system evaluation showed that a decrease in postprandial glucose (PPG) accounted for nearly twice as much as fasting plasma glucose (FPG) for the decreases in HbA1c.^[15]^ So PPG had a better correlation with HbA1c than FPG. The American Diabetes Association Guidelines 2021 suggests that PPG monitoring should be conducted in patients with diabetes who failed to reach satisfactory HbA1c levels but obtained target fasting blood glucose, and PPG levels should be maintained below 10.0 mmol/L to reduce HbA1c.^[16]^ Gastric emptying, intestinal proinsulin system, and preprandial blood glucose levels affect postprandial blood glucose levels. In recent years, more studies have reported that the gut microbiota may exert important actions on blood glucose levels, in addition to the effects of islet functions, diets, exercise, and other factors.^[17,18]^ Studies investigating relationships between diabetes and gut microbiota have successfully shown that gut microbiota composition and abundance are related to fasting blood glucose or HbA1c levels, but few studies have focused on relationships between PPG and the gut microbiota.^[19, 20, 21]^ In this review, we address this issue and examine associated underlying mechanisms.

## Gut microbiota overview

As the second gene bank of human beings,^[22]^ the number of gut microbiota is 10 times the total number of human cells, and the number of genes carried by them is more than 100 times their own. At the same time, as the microbial organ of the host, it is closely related to various metabolic pathways.^[23,24]^ Gut microbiota is established during infancy and develops to maturity across the first 2 years of life. Their stability decreases in one’s old age under the combined influence of genetic and environmental factors. ^[25]^ Gut microbiota can be classified by phylum, class, order, family, genus, and species, and mainly includes *Firmicutes* (*Lactobacillus, Clostridium, Ruminococcus, etc.*), *Bacteroidetes* (*Bacteroides, Prevotella, etc.), Proteobacteria (Escherichia coli, etc.*), and *Actinobacteria* (*Bifidobacteria, etc.*). Firmicutes contain the greatest number of genera, comprising more than 200 genera.^[26]^ Regarded as a unique host microbial organ, intestinal microorganisms are closely associated with different metabolic pathways. Gut microbiota breaks down undigested plant polysaccharides, proteins or amino acids by encoding active enzymes of carbohydrates and protein,^[27]^ and those resultant metabolites could exert a biological activity and affect the metabolism of body.^[28]^

## PPG and its clinical significance

PPG is one of the main reasons for increased HbA1c levels.^[29, 30]^ High PPG is closely related to the occurrence and development of chronic diabetic complications.^[31, 32, 33]^ Hyperglycemia and its effect after acute myocardial infarction on cardiovascular outcomes in patients with type 2 diabetes mellitus (HEART2D) study compared the effects of different blood glucose control strategies on cardiovascular endpoints in 1115 type 2 diabetes (T2D) patients after acute myocardial infarction. The study showed that in patients older than 65.7 years, there is no significant differences in baseline characteristics including HbA1c, diabetic therapies, prior cardiovascular disease history, or other clinically relevant measures between different study arms.^[34]^ The PPG control group recorded a significantly less time to the first cardiovascular event, and a significantly lower proportion of patients experienced a first cardiovascular event when compared with the fasting blood glucose control group (*n* = 56 [29.6%] *vs*. *n* = 85 [40.5%]; hazard ratio = 0.69 [95% confidence interval (CI): 0.49 to 0.96]; *P* = 0.029). Controlling PPG is important for promoting HbA1c levels and preventing microvascular and macrovascular diseases and heart events in diabetes.^[35]^ After eating the same foods, PPG in an Asian population was higher when compared with Caucasians, and was putatively related to chewing habits, basic oral hygiene, high amylase activity,^[36]^ oral physiological, anatomical parameters and other factors in these populations.^[37]^ For these populations, PPG appeared to contribute to HbA1c. Therefore, more attention must be paid to PPG in this group.

## Relationships between gut microbiota and PPG

### In vitro research progress on gut microbiota and glucose lipid metabolism

The molecular mechanisms by which gut microbiota affects host metabolic balance mainly include the following two ways: (1) the role of gut microbiota itself; (2) the effects mediated by metabolites of gut microbiota.^[38,39]^ Gut microbiota can have a direct impact on the host by disrupting the integrity of the intestinal mucosal barrier, allowing molecules such as lipopolysaccharides (LPS) to enter the host cycle. LPS of *Akkermansia muciniphila* can specifically activate the expression of Toll-like receptors 2 (TLR2).^[40]^ A His-tagged Amuc_1100 produced in *E. coli* (hereafter called Amuc_1100*) could similarly signal TLR2-expressing cells to *A. muciniphila*. Plover *et al*. ^[41]^’s research has shown that Amuc_ 1100 * improves the metabolic syndrome in obese and diabetic mice through TLR2 signaling. Compared with normal-fed mice, untreated high-fat fed mice exhibited lower phosphorylation of the protein kinase B (PKB/Akt) pathway, while mice fed with Amuc_1100 * treatment offset this impact and improved insulin sensitivity. In addition, gut microbiota can also have indirect effects through its metabolites, such as the production of short-chain fatty acids (SCFAs), bile acids (BAs), trimethylamine N-oxide (TMAO), *etc*., which affect the metabolism (Table 1).

**Table 1:** The mechanism of the effect of gut microbiota metabolites on postprandial blood glucose

Gut microbiota metabolites	Related signaling pathways/signaling molecules/key enzymes	Microbiota	Mechanism
SCFA (butyrate, formic, acetic, and propionic acids)	FFAR2 FFAR3	*Bacteroidetes Firmicutes Lachnospiraceae Ruminococcus*	SCFA binds to specific transmembrane receptors FFAR2 and FFAR3 stimulates GLP-1 and PYY secretion, inhibits appetite, and reduces PPG.
BA	FXR TGR5	*Clostridium Bacteroides Lactobacillus Bifidobacterium*	BA regulates PPG through signals from multiple parts of the body. Liver BA-FXR promotes glycogen synthesis; Intestinal BA-TGR5 promotes GLP-1 expression and secretion, while BA-FXR inhibits GLP-1 production; BA-TGR5 mediates satiety in the brain and increases energy consumption in skeletal muscle and brown adipose tissue; Pancreatic islet β BA-TGR5 and BA-FXR in cells induce insulin production.
TMAO, TMA	FMO3 PKA IGF-2 PI3K/Akt	*Bacteroidetes, Firmicutes*	The levels of TMAO and TMA increase after meals, which affect the phosphorylation process of PKA and IGF-2, and block the PI3K/Akt insulin signaling pathway to increase PPG.

SCFA: short-chain fatty acid, FFAR2: free fatty acid receptor 2, FFAR3: free fatty acid receptor 3, BA: bile acid, FXR: farnesoid X receptor, TGR5: Takeda G protein receptor 5, TMAO: trimethylamine oxide, TMA: trimethylamine, FMO3: Flavin containing monooxygenase 3, PI3K/Akt: phosphatidylinositol 3-kinase/protein kinase B, IGF-2: insulin-like growth factor 2, PKA: protein kinase A, GLP-1: glucagon-like peptide 1, PPG: postprandial glucose.

### PPG in different populations

Postprandial glucose responses (PPGR) reflect increased areas under blood glucose response curves within 2 hours after eating.^[42]^ Despite eating the same food, the PPGR of different individuals was significantly different, and the level of PPG was also different. In addition to food characteristics (*e.g.* carbohydrate content) and genetic factors, PPGR may be affected by intestinal microbiome differences in different individuals.^[43,44]^

### PPG in non-diabetic individuals

In normal populations, PPG levels may be somewhat predicted by combined information such as diet, body composition, and gut microbiota. Zeevi *et al*.^[45]^ integrated data from 800 non-diabetic subjects and generated a personalized PPGR prediction model, and showed that *Proteobacteria* and *Enterobacter* were positively associated with PPGR in standardized diets. Using a standardized PPGR prediction model in healthy Danish adults, it was suggested that intestinal metagenomic species abundance, specifically, *Clostridia MGS.hg0341* and *Bifidobacteria* were negatively associated with PPG.^[46]^ Nolte *et al*. reported similar findings that *Faecalibacterium prausnitzii* was negatively associated with PPG.^[47]^ In the elderly, certain correlations exist between intestinal bacteria and PPG; research in elderly healthy individuals (> 65 years old) reported significant correlations between gut bacteria and peak glucose levels after dinner and the 4-hour area under the curve (AUC) period after dinner.^[48]^ *Bacteroidetes*, *Blautia*, and *Bilophila* are positive, while *Ruminococcus* and *Holdmannia* are negatively associated with PPG.^[49]^

### Relationships between gut microbiota and diabetic PPG

Intestinal microbial composition in patients with diabetes is distinct when compared with healthy individuals. A systematic review of 25 studies comprising 2209 type 1 diabetes (T1D) and T2D patients reported no significant changes in the number of gut microbiota in this population, but *Bacteroides*, *Bifidobacterium*, and *Clostridium* abundance had decreased and were negatively associated with blood sugar levels.^[50]^

For patients with T1D, decreased *Firmicutes/Bacteroides* ratios may be related to T1D incidences and increased HbA1c levels.^[51]^ Gut microbiota diversity in T1D patients is associated with HbA1c levels.^[52]^ Research has suggested that gut microbiota diversity in adult T1D patients who are not newly diagnosed and whose mean/median HbA1c levels are less than 8% are similar to those of normal individuals, but when HbA1c levels exceed 8%, gut microbiota diversity is distinct from healthy individuals. ^[53]^ A previous T1D study reported that gut microbiota affected PPG; a prediction model incorporated gut microbiota characteristics can predict PPGR in T1D patients, except for the effects of carbohydrate content, and the proportion of carbohydrate to fat on PPG.^[54,55]^ Shilo *et al*. enrolled 121 T1D patients, measured 6377 PPG data points, and designed a prediction model, which integrated blood glucose levels, insulin doses, dietary habits, and gut microbiota to accurately predict PPGR and provide T1D patients with optimal meal insulin doses.^[56]^

For T2D patients, the study showed that *Roseburia* and *Faecalibacterium* abundance decreased, while *Lactobacillus gasseri*, *Streptococcus mutans*, and some *Clostridium spp*. abundance increased when compared with a healthy population.^[57]^ Blood glucose levels in T2D patients are associated with gut microbiota abundance.^[58]^ *Enterobacteria* and *Enterococci* abundance is less in patients with good blood glucose control (HbA1c < 6.5%) when compared with patients with poor blood glucose control (HbA1c ≥ 6.5%), while *Bifidobacteria* and *Bacteroidetes* abundance is higher when compared with patients with poor blood glucose control.^[59]^ Studies have shown that intestinal microbial interventions can affect PPG. In one study, 102 T2D patients were randomly divided into two groups; the control group was given basic hypoglycemic drugs and the intervention group was given hypoglycemic drugs plus triple viable *Bifidobacterium* capsules. After 8 weeks, *Bifidobacteria* and *Lactobacilli* levels increased in the intervention group when compared with the control group, and *Enterococcus* and *coccobacillus* abundance decreased. Also, mean 2-hour PPG levels in the intervention group were 1.54 mmo/L lower when compared with the control group (*P* = 0.026).^[60]^

### How the gut microbiota affects PPG

**Figure 1:**
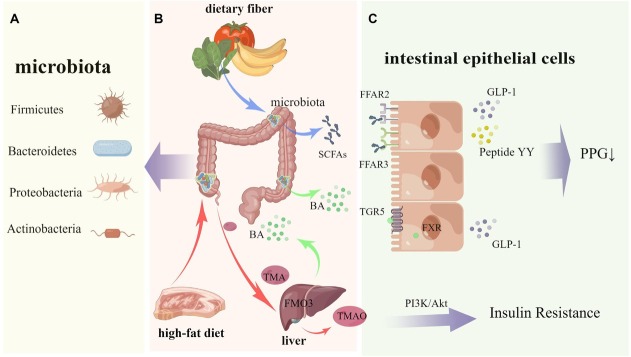
Mechanisms showing how the microbiota potentially reduce postprandial blood glucose. A. Gut microbiota mainly includes Firmicutes, Bacteroidetes, Proteobateria and Actinobacteria. B. Undigested dietary fiber generates SCFAs via microbiota fermentation in the intestine (blue arrow); BA is secreted into bile by liver cells, enters the intestine and participates in the hepatointestinal circulation (green arrow); After high-fat food is eaten, TMA is generated through gut microbiota enzymes, and TMA generates TMAO under the action of liver FMO3 (red arrow). C. Gut microbiota generates many signaling metabolites, such as SCFAs, BAs and TMAO, which participate in different metabolic pathways to ultimately affect PPG. SCFAs: short-chain fatty acids, BA: bile acid, TMA: trimethylamine, FMO3: Flavin containing monooxygenase 3, TMAO: trimethylamine oxide, PI3K/Akt: phosphatidylinositol 3-kinase/protein kinase B, FFAR2: free fatty acid receptor 2, FFAR3: free fatty acid receptor 3, TGR5: Takeda G protein receptor 5, FXR: farnesoid X receptor, GLP-1: glucagon-like peptide 1, PPG: postprandial glucose

In addition to directly acting through its own LPS, gut microbiota can generate various bioactive metabolites such as short-chain fatty acids, bile acids, and trimethylamine oxide through the liver or intestines. These metabolites can serve as indirect regulatory factors, regulating host glucose metabolism and insulin signaling pathways, and affecting postprandial glucose through related metabolic pathways in different tissues and organs (Figure 1).

**Role of short-chain fatty acids:** Undigested dietary fiber generates SCFAs *via* bacterial fermentation in the distal ileum and colon.^[61]^ SCFAs are organic fatty acids that contain 1–6 carbon atoms.^[62]^ Butyrate is the main energy source for gut epithelial cells,^[63]^ and is closely associated with metabolism. SCFAs also include formic, acetic, and propionic acids.^[64]^ Many bacteria produce acetic acid,^[65]^ while *Bacteroidetes* is the dominant propionic acid producer,^[66]^ and *Firmicutes* is the dominant butyrate producer.^[67]^ SCFAs are implicated in carbohydrate metabolism *via* different metabolic pathways to reduce PPG levels.^[68]^ SCFAs and their specific transmembrane receptors, including the free fatty acid receptor 2 (FFAR2) and the free fatty acid receptor 3 (FFAR3), are involved in glucose and lipid metabolism.^[69]^ It was reported that acetic acid selectively mediated Gq/11 or Gi/o pathways *via* FFAR2 and FFAR3 to increase or decrease glucose-induced insulin secretion.^[70]^ FFAR3 has Gi/o coupling, and FFAR2 is doubly coupled through the Gi/o and Gq families.^[71]^ Additionally, butyrate is mainly produced by *Lachnospiraceae* and *Ruminococcus*,^[72]^ which reduces PPG and improves insulin sensitivity *via* epigenetic regulation, mitochondrial β-oxidation, and β-cell proliferation.^[73]^

The gut microbiota impacts PPG by producing SCFAs which stimulate glucagon-like peptide 1 (GLP-1) secretion *via* FFAR2 and FFAR3, induce glucose-dependent insulin secretion and inhibit glucagon secretion.^[74]^ SCFAs also stimulate peptide YY secretion to inhibit the appetite and decrease PPG levels.^[75]^ Vitale *et al*. reported that when compared with control meals, a Mediterranean diet group had significantly lower PPG and insulin responses, and that blood glucose and insulin sensitivity levels were improved after an 8-week dietary intervention.^[53]^ Butyric acid levels in the Mediterranean diet group also increased significantly after meals (*P* = 0.019) and were directly related to insulin sensitivity (*r* = 0.397, *P* = 0.050). These metabolic changes were accompanied by significant changes in intestinal microbiota; when compared with the control group, *Intestinimonas butyriciproducens* and *Akkermansia muciniphila* abundance in the Mediterranean diet group increased.

**Role of bile acids:** BAs are diversified amphipathic steroid molecules which promote intestinal absorption and dietary lipid transportation,^[76]^ with concentrations dependent on biosynthesis, enterohepatic circulation, and intestinal microbiota levels.^[77]^ *Clostridium*, *Bacteroides*, *Lactobacillus*, *Bifidobacterium* and *Enterococcus* have been proven to be involved in the production of bile acid.^[78]^ Recent research reported that BAs are key signal molecules in glucose, lipid, and energy metabolism as they combine with the farnesoid X receptor (FXR) and the Takeda G-protein-coupled receptor 5 (TGR5) in multiple tissues and organs to regulate GLP-1 secretion, gluconeogenesis, glycogen synthesis, inflammatory responses, and gut microbiome structures.^[79, 80, 81, 82]^ FXR is widely expressed in various tissues and organs such as the intestine, liver, and white adipose tissue, which can form heterodimers to inhibit the expression of the rate limiting enzyme cholesterol 7α-hydroxylase (CYP7A1) in BA biosynthesis, weakening cholesterol liver conversion; The expression of CYP7A1 alleviated metabolic disorders associated with obesity, including glucose intolerance, insulin resistance, and dyslipidemia.^[83,84]^ TGR5 is a G protein-coupled receptor, which can produce cAMP through BAs, and then activate the protein kinase A (PKA) pathway.^[85]^ In the liver, BA-FXR signal transduction inhibits gluconeogenesis and promotes glycogen synthesis by negative modulation. In intestinal cells, BA-TGR5 signaling promotes GLP-1 expression and secretion, while BA-FXR signaling inhibits GLP-1 production. In addition, BA-TGR5 signal transduction can mediate satiety in the brain, and increase energy consumption in skeletal muscle and brown adipose tissue. In the pancreas, β BA-TGR5 and BA-FXR signaling in cells induce insulin production.^[86, 87, 88]^ BA sequestrants (BAS) or bariatric surgery can significantly eliminate blood glucose abnormalities. A meta-analysis of 2950 T2D patients from 17 studies reported that HbA1c levels in a BAS group decreased when compared with a control group (mean difference -0.55%; 95% CI: -0.64 to -0.46).^[89]^ Bariatric surgery alters the enterohepatic BA circulation, resulting in increased plasma bile levels as well as altered BA composition.^[90]^ Weight loss surgery, especially Roux-en-Y gastric bypass surgery, can increase circulating BA concentrations.^[91]^ Postoperative BA concentrations are positively correlated with serum GLP-1 concentrations but negatively correlated with PPG.^[92]^ In addition to SCFAs and BAs actions, amino acids and their metabolites, especially tryptophan and associated derivatives, can affect glucose metabolism, but research suggests that amino acid metabolic pathways may affect diabetes and fasting blood glucose levels.^[93, 94, 95]^ However, few studies have investigated the effects of gut microbiota on PPG *via* amino acid pathways.^[96]^

**Effect of trimethylamine oxide:** Ingestion of high-fat foods can generate the primary intestinal metabolite trimethylamine (TMA) through gut microbiota enzymes such as CutC/D,^[97,98]^ CntA/B,^[99]^ and YeaW/X.^[100]^ TMA is mostly produced by *Bacteroidetes* or *Firmicutes* bacteria.^[101]^ Flavin containing monooxygenase 3 (FMO3) in the liver can promote TMA to produce TMAO.^[102,103]^ Previous studies have found that the TMAO pathway is associated with the occurrence and development of diseases such as heart failure, chronic kidney disease, and obesity.^[104,105]^ Research has shown that TMA and TMAO peak levels occur approximately 4 hours after a single feeding of a high-fat diet in fasted mice, indicating that TMA and TMAO are produced after meals and exhibit hormonal oscillations related to TMA source nutrient intake. Some TMA and TMAO are involved in phosphorylation processes such as PKA and insulin like growth factor 2 (IGF-2), and regulate the cascade reaction of insulin signaling.^[106]^ Animal experiments have shown that mice fed a high-fat diet have an increase in TMAO, exacerbating impaired glucose tolerance and insulin resistance, and leading to inflammation of adipose tissue in mice fed a high-fat diet. This process may be related to block the insulin signaling pathway through the phosphatidylinositol 3-kinase/protein kinase B (PI3K/Akt) pathway.^[107]^ An 8-week dietary intervention was conducted on patients with abnormal blood glucose levels, and the results showed a decrease in TMAO levels after intervention with a purely vegetarian diet, accompanied by a decrease in postprandial blood glucose levels.^[108]^ In summary, the gut microbiota can affect PPG through the metabolite TMAO of a high-fat diet.

### Gut microbiota in PPG and its role in precision medicine

Under the combined effects of the intestinal flora and other factors, when the same foods are eaten, blood glucose levels are differentially affected in individuals.^[109]^ The development of individualized hypoglycemic intervention strategies for different blood glucose responses may facilitate more stable blood glucose control strategies. T1D depends on insulin treatment, and appropriate insulin doses are important to control blood glucose levels in patients. The traditional method of calculating good glycemic indices to guide insulin doses is not enough to control T1D blood glucose levels.^[110]^ Based on the tenet that the gut microbiota impacts PPG, prediction models incorporating gut microbiota are important methods for predicting PPGR and may provide personalized treatments for T1D in the future.^[111]^ Individualized nutrition interventions can also affect the gut microbiota and improve PPG. Studies have suggested that environmental factors may have greater roles than genetics in shaping human gut microbiota composition.^[112]^ Personalized nutrition regimens, based on the microbiota, have been used to predict and guide blood glucose levels to generate individualized diabetes prevention and treatment strategies.^[113]^ The cohort study of Zeevi *et al*.^[45]^ monitored the weekly blood glucose level of 800 people and measured the blood glucose response to 46898 meals. The prediction algorithm based on the above data integrates the blood parameters, eating habits, anthropometry, physical activity and intestinal microbiota measured in the queue and can accurately predict the PPGR to the real diet. In addition, they validated these predictions in an independent 100-participant cohort, the authors showed that PPG levels in the bad diet group were significantly higher when compared with the good diet group, and the bad diet group had greater glucose fluctuations evaluated by the continuous blood glucose monitoring system after 1 week. Gut microbiota analyses indicated that *Bifidobacteria* and *Bacteroidetes* abundance were higher in the healthy diet group.

## Conclusion

While numerous studies have shown that gut microbiota is related to PPG and can predict PPGR in non-diabetic populations, limited research focuses on how it predicts and affects PPGR in diabetic patients. More research is required in this area to identify precise interventions and reduce complication risks in diabetic patients. Gut microbiota generates many signaling metabolites, such as SCFAs, BAs, and TMAO, which participate in different metabolic pathways to ultimately affect PPG. However, the precise mechanisms underpinning their impact on PPG remain unclear. Future research must identify key gut microbiota and associated metabolites and pathways impacting PPG and provide potential therapeutic targets for improving PPG outcomes.
